# Death of Dr. Samuel B. Woodward

**Published:** 1850-02

**Authors:** 


Died, at Northampton, in January last, Dr. Samuel B. Woodward, for a long time the Superintendent of the Asylum for the Insane at Worcester, Mass., aged 63. The immediate cause of the death of Dr. Woodward was the rupture of a small aneurismal sac upon the aorta at the point at which it passes through the diaphragm.

